# Two-year randomized clinical trial of enamel matrix derivative treated infrabony defects: radiographic analysis

**DOI:** 10.1186/1472-6831-14-149

**Published:** 2014-12-04

**Authors:** Mariana Schutzer Ragghianti Zangrando, Daniela Chambrone, Ivan Munhoz Pasin, Marina Clemente Conde, Cláudio Mendes Pannuti, Luiz Antônio Pugliesi Alves de Lima

**Affiliations:** School of Dentistry, University of São Paulo, Av. Lineu Prestes, 2227 Cidade Universitária, 05508-000 São Paulo, SP Brazil

**Keywords:** Periodontal regeneration, Enamel matrix derivative, Radiographic analysis, Randomized controlled clinical trial, Infrabony defect

## Abstract

**Background:**

This split-mouth, double-blind randomized controlled trial evaluated radiographic changes in infrabony defects treated with open flap debridement (OFD) or OFD associated with enamel matrix derivative (EMD) after a 24–month follow-up. The radiographic distance from the CEJ to the bottom of the defect (BD) was considered the primary outcome. CEJ-BC and defect angle were secondary outcomes.

**Methods:**

Ten patients presenting 2 or more defects were selected. An individualized film holder was used to take standardized radiographs of the 43 defects, at baseline and after 24 months. Images were digitized and used to measure the distances from the cemento-enamel junction (CEJ) to the alveolar crest (AC), CEJ to the bottom of the defect (BD) and infrabony defect angle. Statistical analysis was performed in SPSS for Windows (version 5.2). Paired samples *t* test was used to compare test and control groups and to evaluate changes within each group. The level of significance was set at α = 0.05%.

**Results:**

After 24 months, a significant crestal bone loss was observed for EMD (1.01 mm; p = 0.049) but not for OFD (0.14 mm; p = 0.622). However, no differences were detected between groups (p = 0.37). Reduction of the bone defect depth was significant for OFD (0.70 mm; p = 0.005) but not for EMD (0.04 mm; p = 0.86), while no differences were detected between them (p = 0.87). Both EMD (0.69°; p = 0.82) and OFD (5.71°; p = 0.24) showed an improvement in defect angle measurements but no significant differences were observed after 24 months or between the groups (p = 0.35).

**Conclusion:**

Linear radiographic analysis was not able to demonstrate superiority of EMD treated infrabony defects when compared to ODF after 24 months.

**Trial registration:**

ClinicalTrials.gov: NCT02195765. Registered 17 July 2014.

## Background

The main goal of periodontal therapy is to halt the destructive process while the ideal objective of periodontal surgery is to regenerate the lost tissues. Regeneration in Periodontics seeks reproduction or reconstitution of a lost or injured part [1], namely formation of new cementum, new alveolar bone, and a functional periodontal ligament.

Different approaches have been proposed to obtain regeneration of periodontal tissues, such as bone grafts, alloplastic materials, guided tissue regeneration, growth factors and enamel matrix derivative (EMD). EMD is known to play a role in the tooth formation, particularly in the formation of acellular cementum [2]. Lately, the use of EMD has been proposed as an alternative in periodontal regeneration. Various studies were performed to evaluate the potential of obtaining periodontal regeneration with this material. *In vitro* studies and animal studies tend to show a benefit on the application of EMD [2–4]. However, prospective controlled clinical trials establishing scientific evidence for the clinical usefulness of EMD are scarce and contradictory [5–14].

Assessment of regenerative procedures is necessary for the evaluation of periodontal therapy. The methods used for evaluation include histology, re-entry surgery, periodontal probing and radiographic analysis [15]. Among those methods, few prospective randomized controlled clinical trials used computerized linear radiographic measurements to evaluate the EMD's application in infrabony defects. Eight clinical trials used clinical and radiographic parameters to evaluate the effect of EMD in infrabony defects [5–9, 11, 13, 14]. Some studies [5–8, 14] observed better results in test groups (EMD) than in control groups (placebo). The EMD groups showed more reduction in probing pocket depth with concomitant gain in periodontal attachment level and increased radiographic bone gain. However, other studies [9, 11, 13] did not demonstrate superiority of EMD in relation to control group. In a recent systematic review [16] about the use of EMD for periodontal tissue regeneration in infrabony defects, only three studies that evaluated radiographic changes were considered at low risk of bias [5, 9, 11]. There was no significant difference between the EMD and the control group regarding radiographic bone gain [16].

The high degree of heterogeneity observed among trials suggests that results have to be interpreted with caution [16]. In light of the available scientific evidence, it is not possible to know the actual clinical advantages of EMD yet [16]. Hence, prospective randomized controlled clinical trials are needed to confirm the clinical and radiographic efficacy of this method for periodontal regeneration.

Thus, the aim of the present study was to compare periodontal radiographic parameters after the treatment of infrabony defects with open flap debridement (OFD) combined or not with enamel matrix derivative (EMD) after 24 months.

## Methods

### Subjects

This split-mouth randomized controlled trial was performed at the Department of Periodontics of the University of São Paulo (São Paulo, Brazil). The project was approved by the Institution's Research Ethics Committee (University of Sao Paulo - School of Dentistry), protocol number 220/03, in accordance with the Helsinki Declaration of 1975, as revised in 2000. Patients were recruited among those who sought periodontal treatment at the Post-graduate Clinic of Periodontics. The following inclusion criteria were considered: (1) diagnosis of chronic periodontitis [17]; (2) presence of at least one pair of interproximal infrabony defects (2–3 walls) adjacent to vital anterior or premolar teeth; (3) absence of degree mobility 3 [18]; (4) probing pocket depth (PPD) ≥ 5 mm; (5) full-mouth plaque score ≤20% [19]; and (6) keratinized tissue width of at least 2 mm. The exclusion criteria were: (1) presence of any systemic disease that could interfere with periodontal treatment; (2) infrabony defects with trans-surgical depth ≤4 mm; (3) antibiotic treatment within the last 6 months. Patients who volunteered to participate in the study gave informed consent and were recruited from June to October 2002.

### Clinical procedures

Following initial examination, all patients were given oral hygiene instruction and full-mouth supra- and sub-gingival scaling and root planning under local anesthesia. Patients were re-evaluated after 4–6 weeks of the initial therapy to determine their response to therapy and to confirm the need for periodontal surgery.

All surgical procedures, until the defects were completely debrided were performed by the same operator (DC). Treatment modality was assigned to each defect by a flip of a coin (MC) to receive either EMD following OFD or OFD alone. Following local anesthesia, all sites were treated with reflection of a full thickness mucoperiosteal flap after intra-sulcular incisions. The exposed roots and osseous defects were debrided with hand instruments, and the surgical wound was rinsed with saline. The first surgeon (DC) left the room in order to keep the study blinded. Another surgeon (MC) treated the selected sites with Prefgel and Emdogain (MC). Both defects were treated at the same surgical time. Adjacent sites were treated by the same therapy while sites localized in the opposite side of the arch were treated differently.

After that, the flaps of the OFD sites were repositioned and sutured using 5–0 nylon sutures (Tech-Lon, TechSynt/Lukens). The EMD sites were dried with non-woven gauze, roots were conditioned with 24% ethylenediaminotetracetic acid (EDTA) gel (pH 6.7; Prefgel, Straumann) for 2 minutes. The defect was thoroughly rinsed with saline, and EMD (Emdogain, Straumann) gel was applied to the root surfaces according to the manufacturer’s instructions. The flaps were then replaced and sutured with 5–0 nylon sutures. The sutures were removed after 7 days.

All patients were prescribed 0.12% chlorhexidine digluconate and instructed to rinse gently twice a day for 4 weeks. Analgesics were prescribed to be taken as needed. All patients returned for professional tooth cleaning once a week, for 8 weeks. Only supragingival instrumentation was performed during supportive periodontal treatment. Subsequently, the patients were maintained in a supportive periodontal program (ie, professional tooth cleaning and reinforcement of self-administered oral hygiene measures) at 2-month intervals up to 6-months and then every 3 months until the final examination at 24 months. Clinical results of the present RCT were presented elsewhere [10, 12].

### Radiographic evaluation

Standardized periapical radiographs were taken at baseline evaluation, immediately before surgery and after 24 months. Individually customized bite blocks employing a reference occlusal stent and film holders were used to obtain reproducible exposed films at each radiographic control. All radiographic images were evaluated by a single examiner blind to treatment and follow-up period (MSRZ). Analyses of the radiographic outcomes were performed using computerized linear measurements with image analysis software (Axiovision v. 3.0; Carl Zeiss). The radiographs were previously scanned in digital format by a scanner (SprintScan 35, Polaroid) at a resolution of 500dpi/8bits. Calibration of the software was achieved with a 1×1 mm radiographic grid. The radiographic analysis was based in anatomical landmarks (CEJ, BD and AC) that were identified on the scanned radiographs.

All linear measurements were recorded by a blinded, calibrated examiner (MSRZ) (intra-class correlation coefficient = 0.98). Each parameter was evaluated using 10 pairs of radiographs (baseline and 24 months) in two stages, with an interval of 15 days between two assessments.

**Figure 1:**
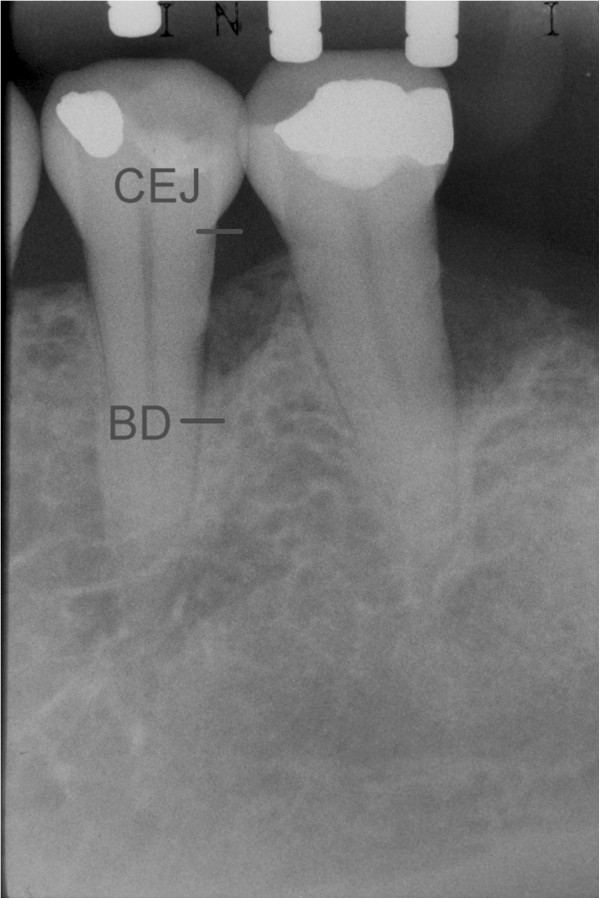
**Distance from CEJ to BD.**

**Figure 2:**
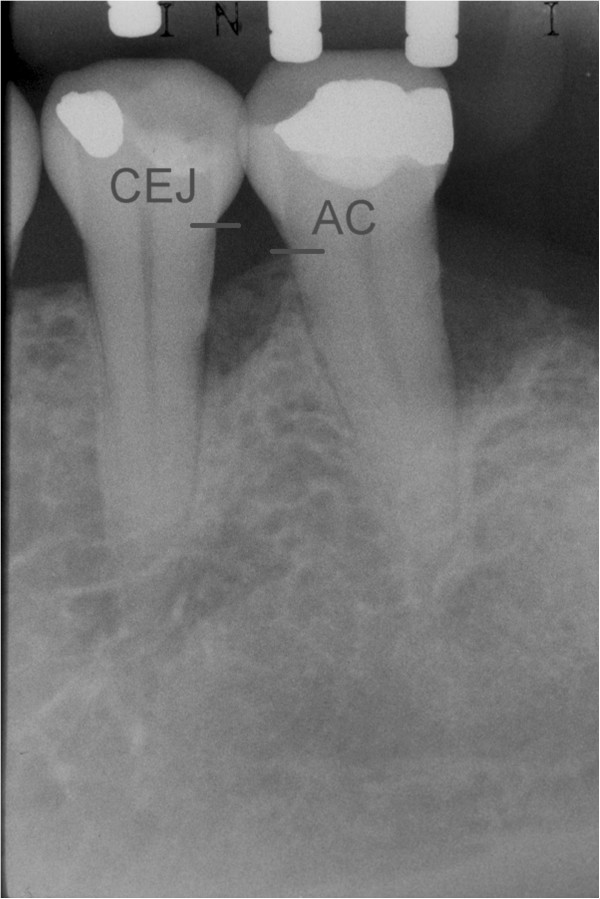
**Distance from CEJ to AC.**

**Figure 3:**
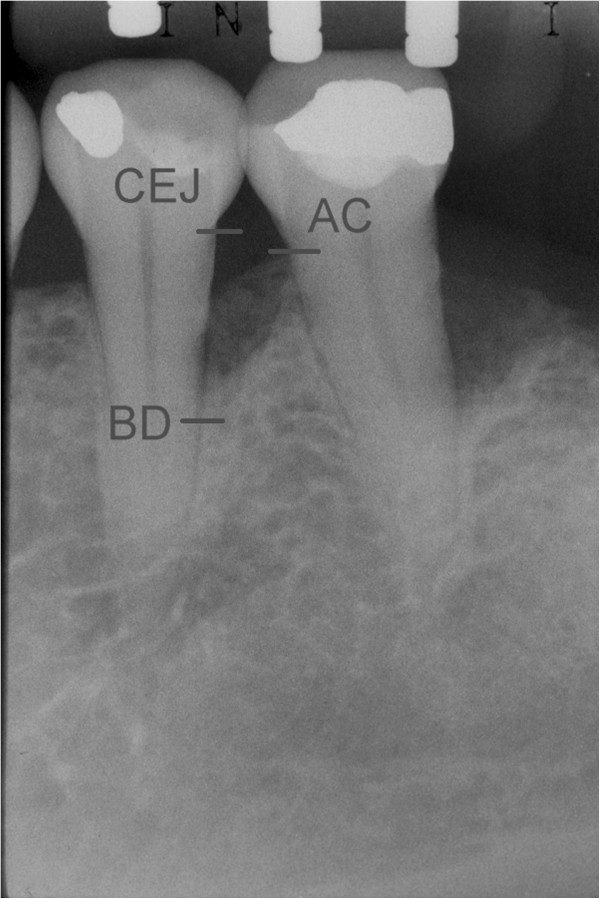
**Angle formed between lines CEJ-BD and CEJ-AC.**

The following outcomes were measured at baseline and after 24 months:

1. Distance from the CEJ to the bottom of the defect (BD). The most coronal area where the periodontal ligament maintained an even width was identified to measure the most apical extension of the infrabony defect [20] (Figure 1);
2. Distance from the CEJ to the bone crest (BC) (Figure 2);
3. Infrabony defect angle was defined by two lines that represented the root surface of the involved tooth (CEJ-BD) and the bone defect surface (BD-BC) [21] (Figure 3).

### Sample size calculation

The distance from the CEJ to the bottom of the defect (BD) was considered the primary outcome. CEJ-BC and defect angle were secondary outcomes. Based on a 0.8 power to detect a significant difference of 2.0 mm in CEJ-BD (α = 0.05; SD = 2.0 mm), 12 volunteers would be required for the trial. Sample size calculation was conducted considering the split-mouth design, in which experimental groups are paired.

### Statistical analysis

Initially, as most patients presented more than one pair of infrabony defects, individual site data were grouped and converted as means of each subject, according to the treatment group. Subsequently, means, medians, and standard deviations for the variables CEJ-BC, CEJ-BD, and defect angle were calculated for both groups (ie, EMD and OFD) using patient as the unit of analysis. Taking into account the nature of the split-mouth design, where both experimental groups are related, paired samples *t* test was used to compare test and control groups and to analyze changes within each group from baseline to the 24-month examination. Adherence to normal distribution was verified using Kolmogorov-Smirnov test. Data had been registered in an elaborated database in Excel (version 7.0). The statistical analysis was performed in SPSS program for Windows (version 5.2). The level of significance was set at α = 0.05%.

## Results

**Figure 4:**
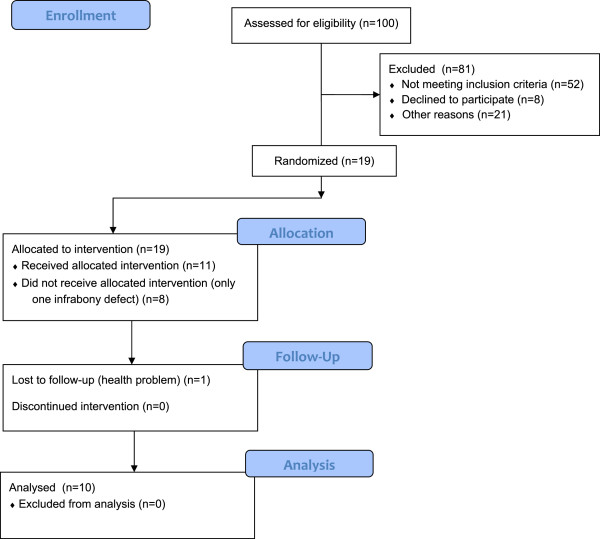
**Flowchart for study patients.**

Figure 4 illustrates the study flowchart according to CONSORT guidelines. At 24 months follow up, 10 patients (two men and eight women; three smokers and seven non-smokers) 28 to 50 years old (38.8 ± 5.7), with 43 infrabony defects (ie, 18 defects in the control group and 25 in the test group) were analyzed for the primary and secondary outcomes.

Postoperative healing was uneventful in all cases, and no complications or adverse effects were observed throughout the study period.

Table 1 reports the mean and standard deviations of the distance CEJ-AC and CEJ-BD for experimental groups at baseline and after 24 months. There were no differences in CEJ-AC values between groups at baseline. After 24 months, a significant crestal bone loss (CEJ-AC) was observed for EMD (1.01 mm; p = 0.049) but not for OFD (0.14 mm; p = 0.622). However, no differences were detected between groups (p = 0.37). At baseline, mean value of CEJ-BD was 10.11 for test group and 8.67 for control, with no statistical difference between groups. After 24 months, reduction of the bone defect depth (CEJ-BD) was significant for OFD (0.70 mm; p = 0.005) but not for EMD (-0.04 mm; p = 0.86), while no differences were detected between them (p = 0.87).

**Table 1:** **Mean, standard deviation and comparison of experimental groups for CEJ-AC and CEJ-BD (N = 10 patients)**

Time period	Mean/SD	EMD (mm)	OFD (mm)	Paired ***t***test
Baseline	Mean	5.13	5.10	0.35
CEJ-AC	SD	2.59	2.31	
24 months	Mean	6.14	5.24	0.37
CEJ-AC	SD	2.47	2.73	
	Paired *t* test	0.049*	0.622	
Baseline	Mean	10.11	8.67	0.93
CEJ-BD	SD	2.63	2.43	
24 months	Mean	10.15	7.97	0.87
CEJ-BD	SD	2.78	2.29	
	Paired *t* test	0.86	0.005*	

SD = standard deviation, *statistical significance 0.05%.

Table 2 shows the mean and median values and standard deviations of defect angle for experimental groups at baseline and after 24 months. At baseline, the mean value of defect angle was the same for test and control group (29.64°). Both EMD (0.69°; p = 0.82) and OFD (5.71°; p = 0.24) showed an improvement in defect angle measurements but no significant differences were observed after 24 months or between them (p = 0.35).

**Table 2:** **Mean, median, standard deviation and comparison of experimental groups for defect angle (N = 10 patients)**

Time period	Mean/SD	EMD (mm)	OFD (mm)	Paired ***t***test
Baseline	Mean	29.64	29.64	0.99
	Median	25.99	32.71	
	SD	9.65	12.39	
24 months	Mean	30.33	35.35	0.35
	Median	27.05	34.30	
	SD	11.21	16.53	
	Paired *t* test	0.82	0.24	

SD = standard deviation.

## Discussion

After 24 months, inter-group comparisons (EMD versus OFD) showed that the use of EMD did not present additional benefits in radiographic measurements. Reduction of the bone defect depth was significant in OFD group (0.7 mm; p = 0.005) but not for the test group (-0.04 mm; p = 0.86). However, no differences were detected between groups for both parameters. In accordance with the present results, other investigators which evaluated radiographic measurements did not find differences between groups after 12 months (EMD = 1.55 mm; Placebo = 1.39 mm, p = 0.9) [9], (EMD = 2.5 mm; Placebo = 2.5 mm, p = 0.81) [11]. Nevertheless, previous clinical trials showed greater mean radiographic bone gain for EMD group: 2.6 mm [5], 2.4 mm [6] and 3.44 mm [8]. Regarding the crestal bone, there was significant crestal bone loss in the test group (1.01 mm; p = 0.049) but not for the control group (0.14 mm; p = 0.622) with no differences between groups. A limitation of the present trial was the reduced sample size and consequent low power to detect differences between groups. In agreement with the results of this study, other trials [9, 11, 13] did not find differences between groups. Results of this study regarding angle of the defects have shown that at baseline, mean values of defect angle were the same (29.64°) for both groups. Both EMD (0.69°; p = 0.82) and OFD (5.71°; p = 0.24) showed an improvement in defect angle measurements but no significant differences were observed after 24 months or between them (p = 0.35). In this study, EMD was not able to produce additional radiographic benefits compared with the OFD alone. In contrast, Francetti et al. [8] observed that in EMD group, infrabony defect angle (IBA) at either 12 (50.2°) or 24 months (51.7°) was significantly increased when compared with baseline (31.7°). In the control group, IBA did not show significant changes at 12 (40.2°) and 24 months (41.3°) with respect to baseline (32.5°). A significant difference between groups was found for the 12-month but not for the 24-month follow-up. According to Steffensen and Weber [21] the presence of a defect angle lower than 45° would be more favorable to radiographic bone healing. Although defect angle was lower than 45° in the present study. we could not observe a favorable result. These results might be justified because of the inherent variability of results achieved with EMD technique [16] or due to the fact that three of our subjects were smokers [22].

Clinical parameters (relative attachment level-RAL, periodontal probing depth-PPD, plaque index-PI and gingival index-GI) were evaluated at 6, 12, 18 and 24 months of follow up [10, 12]. Both procedures (EMD and OFD) were effective in reducing PPD, with concomitant gain in RAL (p < 0.05). Compared with baseline data, the 24-month examination showed a mean reduction in PPD of 4.21 ± 0.97 mm for the test group and 3.28 ± 1.23 mm for the control group. The reduction was statistically significant for EMD group. Mean gain in RAL was 5.69 ± 1.96 mm for test group and 5.24 ± 1.55 mm for control group. However, no differences were detected between groups. Both groups presented a significant reduction of PI and GI throughout the experiment. Although favorable clinical results were achieved for both groups, none of the two above clinical parameters represent or correlate to bone levels. These aspects stress the importance of radiographic evaluation of regenerative procedures and justify the results of the present study due to the fact that the clinical improvement achieved with EMD and OFD may not be expressed to the same extent on bone levels. Studies [23, 24] evaluating periodontal regeneration also found no correlation between clinical parameters and radiographic bone linear measurements or subtraction analysis.

According to Zanatta et al. [25], it is evident that clinical and radiographic methods are not safe in the determination of the healing pattern that occurs after regenerative therapy. Thus, *in vitro* studies support the concept that EMD may enhance periodontal regeneration with formation of acellular cementum and stimulation of periodontal ligament cells [2, 26]. Conversely, EMD effects on osteoblastic cells were variable according to the cell species and/or culture conditions [27]. Hama et al. [27] reported that EMD may function initially to inhibit osteoblastic differentiation to allow a predominant formation of other periodontal tissues. Plachokova et al. [28] concluded that Emdogain is not osteoinductive and does not provide an additional stimulus for bone formation. This *in vivo* study demonstrated that use of EMD for the purpose of generating new bone in clinical situations should be questioned. Windisch et al. [29] observed less bone formation for EMD compared to guided tissue regeneration group, but with improvement of clinical parameters in both groups. Even in the absence of bone, the presence of periodontal ligament cells is sufficient for fibrous reattachment between root surface and the surrounding tissue [30]. These findings are also important from a clinical point of view, since they indicate that the absence of a radiographic defect fill does not necessarily imply in failure of the therapy [29]. Based on a previously published study [12] with favorable clinical outcomes in this sample, it is possible to infer that clinical attachment gain occurred, but without defect bone fill.

As a matter of fact, the present study showed lower mean values of radiographic bone fill compared with the literature. Nevertheless, it is important to report that out of 43 bone defects, 60.46% presented bone gain (CEJ-BD), 46.15% (mean gain = 1.3 mm) from test group and 53.85% (mean gain = 1.04 mm) from control. Bone loss occurred in 37.2% sites, being 75% (mean loss = 1.06 mm) in test sites and 25% (mean loss = 0.49 mm) in control sites.

The variability of results obtained in different trials might be related to diverse aspects such as the place where the study was conducted, inclusion of smokers, type of infrabony defects treated, type of periodontal disease, persistence of specific periodontal pathogens, differences in the technical ability and experience of the clinician and capacity of clinical organization and data collection [31], use of placebo, antibiotics, differences in surgical techniques and root conditioning [16].

The small sample size and inclusion of smokers might have contributed for the unfavorable results observed in the present study. Tobacco smoking is known to produce negative effects on periodontal regenerative therapy [22, 32, 33]. Two clinical trials [11, 13] reported lack of a statistical difference between EMD and placebo-treated sites. One [11] attributed part of this outcome to tobacco use, since there were more heavy smokers in the test group, although the other trial [13] excluded smokers.

A systematic review and meta-analysis [25] suggested that the magnitude of differences between the use of EMD and OFD considerably decreases over time, when studies with follow-up of 12 months and follow up ≥24 months are compared. Considering the quality of the included studies, those with low risk of bias showed lower differences between groups. In the systematic review by Esposito et al. [16], out of 35 studies, only three trials were selected for radiographic analysis [5, 9, 11]. This findings stress the importance of producing more data on radiographic analysis of EMD. Meta-analysis of nine trials demonstrated that the application of EMD showed improvements in periodontal attachment level (PAL = 1.1 mm) and PPD reduction (PPD = 0.9 mm). While the improvements in PAL and PPD levels were positive findings, the real clinical utility of EMD was debated. In particular, it was reported that there is no evidence that more compromised teeth could be saved or the amount of tissue regeneration is clinically significant [16].

With respect to generalization of the findings, in this study, treatments were administered by an experienced clinician which is not always the case in a clinical setting. Moreover, a very strict maintenance regimen was adopted, which also is not generally a routine in clinical situations. Even considering these restrictive conditions, the results presented high variability and low clinical significance [16].

## Conclusions

Although the application of EMD for periodontal regeneration may result in favorable clinical outcomes, we did not observe an additional benefit on bone gain. Linear radiographic analysis was not able to demonstrate superiority of EMD treated infrabony defects when compared to ODF after 24 months. Evidences of the efficacy of EMD on the treatment of infrabony defects are conflicting. More randomized controlled clinical trials, with low risk of bias and larger samples, are necessary to further investigate if there are actual clinical and radiographic advantages of using EMD for periodontal regenerative therapy.

## Authors’ information

MSRZ- Assistant Professor, Division of Periodontics, Department of Prosthodontics, Bauru School of Dentistry, University of Sao Paulo, Bauru-SP, Brazil.

DC- PhD in Periodontics, private practice.

IMP- MSc in Periodontics, private practice.

MCC- Assistant Professor, Division of Periodontics, Department of Stomatology, School of Dentistry, University of Sao Paulo, São Paulo, Brazil.

CMP- Assistant Professor, Division of Periodontics, Department of Stomatology, School of Dentistry, University of Sao Paulo, São Paulo, Brazil.

LAPAL - Associate Professor, Division of Periodontics, Department of Stomatology, School of Dentistry, University of Sao Paulo, São Paulo, Brazil.

## Abbreviations

OFD: Open flap debridement
EMD: Enamel matrix derivative
CEJ: Cemento-enamel junction
AC: Alveolar crest
BD: Bottom of the defect
IBA: Infrabony defect angle
RAL: Relative attachment level
PPD: Periodontal probing depth
PI: Plaque index
GI: Gingival index.
